# Research priorities for intra-articular corticosteroid injections for osteoarthritis: A Delphi study

**DOI:** 10.1016/j.ocarto.2022.100291

**Published:** 2022-09

**Authors:** Vikki Wylde, Andrew J. Moore, Edith Anderson, Richard Donovan, Ashley W. Blom, Andrew Judge, Michael R. Whitehouse

**Affiliations:** a Musculoskeletal Research Unit, Bristol Medical School, University of Bristol, Bristol, UK; b Bristol Biomedical Research Centre, University Hospitals Bristol and Weston NHS Foundation Trust and the University of Bristol, UK; c Patient Experience Partnership in Research Group, Musculoskeletal Research Unit, Bristol Medical School, University of Bristol, Bristol, UK

**Keywords:** Osteoarthritis, Corticosteroid injections, Research priorities, Delphi

## Abstract

**Objective:**

To identify research priorities for intra-articular corticosteroid injections for osteoarthritis using a Delphi study.

**Design:**

In the Round 1 questionnaire, participants generated up to five potential research topics related to corticosteroid injections for osteoarthritis. These responses were collated and grouped to develop candidate research questions. Literature searches were conducted and questions with a lack of evidence were included in the next round. In Round 2, importance ratings (1–9; not important to very important) were assigned to each question. Those questions given an importance rating of 7–9 by ≥ 70% of participants were carried forward. In Round 3, participants were provided with the group ratings and the rating process was repeated to develop the final research priority list.

**Results:**

All three Delphi rounds were completed by 75 participants (82%; 34 patients, 21 healthcare professionals and 20 academics). A total of 310 research topics were generated in Round 1, from which 26 research questions were developed. None had been robustly answered by research and therefore all were included in the Round 2 questionnaire. In Round 2, 14 research questions were retained; all 14 were prioritised in Round 3 and included in the final research priority list. The questions covered long-term effects, clinical and cost-effectiveness, measurement of outcomes, comparison to other treatments, provision, safety, identifying responders, maximising benefits, patient experience, delaying the need for joint replacement, and dosage.

**Conclusion:**

Using a robust consensus technique with key stakeholders, we have developed a research priority list to guide future research into corticosteroid injections for osteoarthritis.

## 1 Introduction

In the United Kingdom (UK), people with osteoarthritis are initially provided with management through primary healthcare services. Management involves core treatments such as education and advice, exercise, weight loss if appropriate, and use of assistive devices/aids, physical therapy (physiotherapy, insoles, or braces) and pharmacotherapy (paracetamol and non-steroidal anti-inflammatory drugs as first line treatment for pain). Some patients proceed to secondary care management in which there are more invasive treatment options, including joint replacement.

The National Institute for Health and Care Excellence (NICE) Clinical Guideline for osteoarthritis recommends the use of intra-articular corticosteroid injections when other pharmacological treatments are ineffective or unsuitable [1]. Since the publication of the NICE guidance, further reports on the benefits of intra-articular corticosteroid injections for osteoarthritis management have been published [2–5]. The overall evidence from these further findings suggests a short-term benefit of intra-articular corticosteroids on pain relief and mild or no evidence of adverse effects, although the long-term term benefits and risks are unclear [6–8]. There are further uncertainties regarding current practice and patterns of use of intra-articular corticosteroid injections in the UK and globally is also limited [9,10].

Given that the prevalence of osteoarthritis is expected to rise over the coming years and there will likely be an associated rise in the use of intraarticular corticosteroid injections, future research is needed to provide a robust evidence-base that can be used to guide treatment provision and optimise patients’ experiences and outcomes of this procedure. To direct this research, identification of research priorities that reflect the needs and opinions of key stakeholders, including patients, healthcare professionals and academics, is warranted. As part of a programme of work commissioned by the National Institute of Health Research Health Technology Assessment panel (the RUBICON programme, funder reference NIHR129011), the aim of this study was to use a Delphi study to gain expert consensus on future research priorities for intra-articular corticosteroid injections for osteoarthritis and any feasibility considerations associated with future primary research.

## 2 Methods

Ethics approval was obtained from the North of Scotland National Health Service (NHS) Research Ethics Service (21/NS/0070) on the May 24, 2021 and Health Research Authority approval on the May 25, 2021. All participants provided informed, written consent. Recruitment and the three rounds of Delphi questionnaires were completed over an 8-month period between June 2021 and January 2022.

### 2.1 Delphi technique and sample size

The Delphi survey technique is a structured and iterative technique that uses a series of sequential questionnaires completed anonymously by participants with relevant expertise to reach consensus amongst experts about a particular issue [11]. The technique has been used extensively to identify research priorities in a wide range of healthcare settings, with recent examples including malignant oesophagogastric surgery [12], major trauma [13], atypical anorexia nervosa [14] and vascular surgery [15]. In Round 1, open-ended questions are used to elicit information to be used in subsequent rounds. Items generated from Round 1 are then ranked by participants in Round 2 and those items meeting predefined criteria are carried forward to Round 3. A key methodological feature is the use of a staged approach which provides participants with the opportunity to review group feedback and revise their own views. It has several advantages over other methods of gaining consensus, for example, the influence of group dynamics and peer influence are removed as the participants do not interact with one another and remain anonymous to one another [11,16].

The sample size for a Delphi survey should reflect adequate incorporation of stakeholder diversity in obtaining consensus on a topic, rather than statistical power [11]. There is no set recommendations on how many participants should be included in a Delphi survey [16], although a minimum of 10 participants on a panel has been suggested [11]. To ensure the views of all key stakeholders were included in our study, we aimed to recruit a minimum of 75 participants onto three panels: 25 patients, 25 healthcare professionals and 25 academics. The inclusion of different panels in the research design ensured that the views of different key stakeholders were represented in the final consensus [16].

### 2.2 Patient and public involvement (PPI)

This study was designed and conducted in collaboration with members of the Patient Experience Partnership in Research (PEP-R) group [17], which comprises nine patients, all with experience of musculoskeletal conditions. Co-author and research team member ED is a patient with experience of receiving treatment for osteoarthritis. Patient representatives were involved in the research design, and co-producing study documents, plain language wording for the candidate research questions and feedback for participants.

### 2.3 Recruitment

Potential participants were sent study information including an invitation letter and participant information booklet. People interested in participating were asked to complete the Round 1 questionnaire and consent form. Patient participants were given the option to complete the questionnaires on paper or online (via Online Surveys: https://www.onlinesurveys.ac.uk/) and professionals were provided with online questionnaires only.

Patient panel: Eligibility criteria included adults registered with a participating General Practice (GP) surgery who had received one or more intra-articular corticosteroid injections for osteoarthritis in a primary care setting in the past three years. Patients were recruited from three GP surgeries in the Southwest of England via the Clinical Research Network. In addition, patients who met the eligibility criteria and had agreed to be contacted about future research after participating in a qualitative interview as part of the wider RUBICON programme were sent the study information and invited to participate.

Healthcare professional panel: Eligibility criteria included primary and secondary care healthcare professionals (GPs, physiotherapists, rheumatologists, orthopaedic surgeons, and commissioners) with experience of providing care in a clinical capacity for patients receiving intraarticular corticosteroid injections for osteoarthritis or commissioning of musculoskeletal services. Healthcare professionals were recruited through Clinical Research Network networks across the UK, professional organisations and previous research collaboratives. In addition, primary care staff were recruited from three GP surgeries in the Southwest of England via the Clinical Research Network.

Academic panel: Eligibility criteria included academics in the UK who had published research on treatments or care pathways for osteoarthritis. Academics were identified from non-systematic searches of the published literature and e-mailed study information by the research team.

### 2.4 Round 1

In the Round 1 questionnaire, participants were asked to provide some sociodemographic/professional information and provide up to five potential research topics related to intra-articular injections of corticosteroid in osteoarthritis. For each topic generated, participants were asked to reflect if there were likely to be any associated feasibility considerations with conducting research on the topic and to provide details in a free-text field. Participants were asked to provide suggestions for feasibility considerations in the Round 1 questionnaire only to minimise participant burden and optimise retention between Delphi Rounds. The questionnaire for professionals and patients had similar content, but the questionnaire sent to patients was written in plain English, developed in collaboration with patient representatives.

#### 2.4.1 Round 1 analysis

Responses were collated in Excel and research suggestions were grouped by topic and topics reviewed to develop candidate research questions and associated feasibility considerations. Grouping of topics and development of research questions and feasibility considerations was initially conducted by VW and then reviewed and refined by MW. The list of candidate research questions was reviewed by patient representatives who worked with the research team to write the research questions in plain English.

#### 2.4.2 Literature searches

Literature searches were conducted to evaluate if any of the questions identified in Round 1 had been fully answered by existing research to ensure that only questions with a lack of evidence or treatment uncertainty were included in Round 2 (search terms provided in Supplementary materials Fig. 1). Records were screened to remove clearly irrelevant records by one reviewer (RD), and potentially relevant records were screened by a reviewer (VW or RD) and either assigned to the relevant research question or classed as non-relevant. The volume of studies identified that addressed each research question was then categorised as ‘none/low (<10 studies)’, ‘some’ (10–30 studies) or ‘high’ (>30 studies). Research questions with ‘none/low’ studies were classified as not fully answered because of a lack of research. For those candidate research questions with some or a high volume of studies, the existing literature was narratively reviewed by one reviewer and an assessment made on whether the research question had been fully answered based on the scope of the existing literature.

### 2.5 Round 2

Participants who completed a Round 1 questionnaire were invited to participate in Round 2. In the Round 2 questionnaire, participants were asked to rate the importance of each of the candidate research questions from 1 to 9 (not important to very important). A single reminder was sent if no response was received within two weeks.

#### 2.5.1 Round 2 questionnaire analysis

Median scores for each candidate research question were calculated. The criteria for retraining research questions was defined a priori in the study protocol and was based on the RAND/UCLA Appropriateness Method [18]. A research question with a median score of 1–3 was defined as representing limited importance, 4–6 as important but not critical and 7–9 as critically important. Those research questions given an importance rating of 7–9 by ≥ 70% of participants were retained and carried forward to Round 3. To ensure the views of the individual panels were represented in the final consensus, research questions given an importance rating of 7–9 by ≥ 90% of members of one panel, regardless of the ratings of the other panels, were also carried forward.

### 2.6 Round 3

Participants who responded to Round 2 were invited to participate in Round 3. In this final Round, participants were sent a shortened Round 3 questionnaire which contained the research questions retained from Round 2. Participants were also provided with the median group rating and their own individual rating for each research question retained from Round 2. After reviewing the ratings, participants were given the opportunity to re-rate the importance of each research question. A single reminder was sent if no response was received within two weeks.

#### 2.6.1 Round 3 questionnaire analysis

Those questions given an importance rating of 7–9 by ≥ 70% of participants, or by ≥ 90% of members of one panel, were included in the final research priority list.

## 3 Results

### 3.1 Participants

A total of 91 participants were recruited and completed Round 1; 41 patients, 25 health professionals, and 25 academics. Of these participants, 82 (90%; 38 patients, 23 healthcare professionals and 21 academics) completed the Round 2 questionnaire and 75 (82%; 34 patients, 21 healthcare professionals and 20 academics) completed all three Delphi rounds. Retention rates between Delphi rounds were similar across the different panels. Details of recruited participants and those that completed all three Delphi rounds are provided in Table 1.

### 3.2 Round 1

A total of 310 research topics were generated by the 91 participants who completed the Round 1 questionnaire. Twenty-five of these were deemed beyond the scope of this study or not a research question (details provided in Supplementary Table 1); the remaining 285 research topics were coded, from which 26 research questions were developed. Further details are provided in the Supplementary materials Table 2. Feasibility considerations that were raised by participants are described in Table 2. These related to recruitment, long-term follow-up, use of placebo, outcomes assessment, rare occurrence of adverse events, and variability of treatment provision and care pathways.

#### 3.2.1 Literature review

Searches identified 3200 records, and 273 records were identified as potentially relevant to the research questions. The review of the literature revealed that none of the research questions had been robustly answered by previous research (further details provided in the Supplementary materials Table 3) and therefore all 26 research questions were included in the Round 2 questionnaire.

### 3.3 Round 2

Of the 26 research questions included in the Round 2 questionnaire, 14 were given a rating of 7–9 by ≥ 70% participants and carried forward to Round 3. Details of the overall ratings and individual panel ratings are provided in Table 3. Within the individual panels, there was a general tendency for the patient panel to give higher importance ratings to research questions than the other two panels; 22 research questions were given importance ratings of 7–9 by ≥ 70% participants on the patient panel, this reduced to 13 research questions for the healthcare professional panel and four research questions for the academic panel. Only one research question was rated as 7–9 by ≥ 90% participants on a panel; this was “What outcomes are important to patients?”, which was rated as important by 92% of the patient panel. Of the 12 research questions which were not retained and carried forward to Round 3, eight were rated as 7–9 by ≥ 70% of the patient panel, three by the healthcare professional panel and none by the academic panel.

### 3.4 Round 3

After re-rating of each research question by participants considering the average panel ratings, all 14 research questions were given a rating of 7–9 by ≥ 70% participants (Table 2). There was a trend for the percentage of participants giving an importance rating of 7–9 to increase between Rounds 2 and 3. Across the panels there were seven research questions for which one or two panels had <70% of participants assign an importance rating of 7–9; however higher ratings from the other panel(s) resulted in the research question being included in the final research priority list. The 14 prioritised research questions about intraarticular corticosteroid injections are provided in Table 4.

## 4 Discussion

Using a Delphi methodology, we have established 14 research questions for intra-articular corticosteroid injections for osteoarthritis which were collectively prioritised as important by patients, healthcare professionals and academics in the UK. The remit of the research priorities is broad, including clinical and cost-effectiveness, measurement of outcomes, comparison to other treatments, provision, safety, identifying responders, maximising benefits and patient experience. The generation of research priorities that are important to key stakeholders can help to guide future research, ensuring that it reflects the views of patients and professionals working in the area. These research priorities can be used by funders to inform future funding calls, by journals to develop special issue calls and by researchers to focus research efforts on those areas most important to stakeholders.

There were differing views on the importance of the research questions by healthcare professionals, academics and patients. There was a general trend for the patient panel to give higher average importance ratings to the research questions than the other panels. There were also differences in the research questions generated in Round 1 by the different panels; for example, only patient participants suggested questions related to follow-up and increasing the duration of benefit from corticosteroid injections. This emphasises the importance of involving patients in this process; in a patient-centred healthcare system, patient voices must be central to identifying and prioritising areas for research. In our study patients were well represented in the Delphi process, with more patient participants than academic or healthcare professional participants. However, in other studies developing research priorities there has been low or no representation from patients [12,13,15]. In our study, PPI in the study design and co-working with patient representatives to develop the study documents was invaluable to ensure that the language and approaches used were accessible to patient participants.

This study has a number of strengths and limitations which should be acknowledged when interpreting the results. We used a robust and structured process for gaining consensus and applied pre-specified criteria for prioritising the research questions. Participant retention between Delphi rounds was high (82% of recruited participants completed all three rounds) and retention was similar between the three panels, reflecting ongoing engagement by all members of all participant panels in the process. However, we acknowledge that other methods can be used to develop research priorities. For example, The James Lind Alliance Priority Setting Partnerships are collaborative initiatives that identify and prioritise evidence uncertainties in specific conditions or healthcare settings that could be answered by research, for example early hip and knee osteoarthritis [19] and problematic knee replacement [20]. However, Priority Setting Partnerships can be costly, complex and have a long duration; the Delphi approach offered a cost-effective approach to the timely generation of research priorities.

Participants in our study reflected the key stakeholders relevant to the topic, ensuring that the research priorities are relevant to those receiving, administrating, commissioning and researching corticosteroid injections. The multidisciplinary composition of both the healthcare professional and academic panels ensured the inclusion of diversity of views in the consensus process. However, our patient panel lacked ethnic diversity; this may be a reflection that our recruitment was limited to a small number of GP surgeries in the South West of England and did not explore other avenues to reach underserved communities, for example through engagement with faith organisations or local community groups. Also our focus was on developing research priorities relevant to the NHS and therefore our priority list does not reflect the views of people from outside the UK. We had broad inclusion criteria for the academic panel and therefore participants may have had limited expertise in the field of intra-articular corticosteroid injections. Finally, our process for determining if the candidate research questions had been fully answered by previous research was limited as it was not conducted in duplicate and there was no formal assessment of study quality.

In conclusion, the generation of these research priorities can be used by funding bodies and researchers to shape the research landscape and ensure that future research reflects the priorities of key stakeholders. By informing the research agenda, they will promote the development of research that is directed towards the key unanswered questions that could lead to improved care and outcomes for patients receiving intraarticular corticosteroid injections.

## Supplementary Material

Supplementary data to this article can be found online at https://doi.org/10.1016/j.ocarto.2022.100291.

Supplementary Material

## Figures and Tables

**Table 1:** Participant characteristics.

	Recruited	Completed study
Patient panel		
Number	41	34
Median age in years (range)	75 (51–87)	75 (51–87)
Gender (women:men)	27:14	22:12
Ethnic group: White	41	34
**Healthcare professional panel**		
Number	25	21
Gender (women:men)	9:16	8:13
Mean years of experience in profession (standard deviation)	18 [10]	17 [10]
Profession (number)		
GP	10	9
Physiotherapist	7	6
Orthopaedic surgeon	4	3
Rheumatologist	2	2
Commissioner	2	1
Region (number)		
South West of England	13	12
North East of England	6	4
South East of England	3	2
East Midlands	1	1
West Midlands	1	1
London	1	1
**Academic panel**		
Number	25	20
Gender (women:men)	8:17	8:12
Mean years of experience (standard deviation)	20 [12]	22 [12]
Profession (number)	
Academic orthopaedic surgeon	9	6
Academic physiotherapist	8	7
Academic rheumatologist	2	2
Health services researcher	2	1
Academic GP	1	1
Research nurse	1	1
Health economist	1	1
Statistician	1	1
Region (number)		
South West of England	11	9
West Midlands of England	5	4
South East of England	4	3
Scotland	3	2
North West of England	1	1
East of England	1	1

GP = General Practitioner.

**Table 2:** Feasibility considerations associated with the candidate research questions from the Round 1 questionnaire.

Feasibility consideration	Relevant research question
Corticosteroid injections can be administered in GP practices and in hospitals, and research studies would need to recruit patients from both settings	General
Long-term follow may be difficult if patients with persisting symptoms receive joint replacement	General
Would patients agree to take part in a trial comparing a real corticosteroid injection with a placebo corticosteroid injection?	Do corticosteroid injections reduce osteoarthritis symptoms?
Would it be ethical to give patients a placebo injection?	Do corticosteroid injections reduce osteoarthritis symptoms?
Could all the effects of corticosteroid injections be measured within a trial?	Do corticosteroid injections reduce osteoarthritis symptoms?
There are many different factors that might predict how much people benefit from corticosteroid injections	Is it possible to predict which patients are most likely to benefit from corticosteroid injections?
Adverse effects from corticosteroid injections are rare and so a large study would be required	What are the risks of corticosteroid injections?
	How many corticosteroid injections is it safe for patients to receive?
Treatment pathways can be variable	When in the osteoarthritis treatment pathway should patients be offered corticosteroid injections?
The physiotherapy that patients receive can be variable	Do corticosteroid injections help people to do exercises/physiotherapy?
Challenges of recruiting patients from diverse backgrounds	Is there fair access for patients to corticosteroid injections?

**Table 3:** Delphi Round 2 and 3 results.

Research question	% 7–9 rating							
	Overall		Patient panel		Health professional panel		Academic panel	
	R2	R3	R2	R3	R2	R3	R2	R3
What are the long-term effects of repeated intra-articular corticosteroid injections for osteoarthritis?	80	89	81	91	82	90	76	85
What outcomes are important to patients having intra-articular corticosteroid injections for osteoarthritis?	80	85	92	94	65	71	76	85
Are intra-articular corticosteroid injections as good as other non-surgical treatments at reducing osteoarthritis symptoms?	72	84	73	85	74	81	67	85
Can the duration of benefit from intra-articular corticosteroid injections for osteoarthritis be increased?	73	80	86	91	57	76	67	65
When in the osteoarthritis treatment pathway should patients be offered intra-articular corticosteroid injections?	77	80	83	94	77	62	67	75
How many intra-articular corticosteroid injections is it safe for patients with osteoarthritis to receive?	76	80	78	79	86	76	62	85
Do intra-articular corticosteroid injections reduce osteoarthritis symptoms?	70	79	74	82	70	67	62	85
Are intra-articular corticosteroid injections for osteoarthritis good value for money for the NHS?	74	77	76	76	74	81	71	75
Is it possible to predict which patients are most likely to benefit from intra-articular corticosteroid injections for osteoarthritis?	72	77	68	71	83	81	67	85
Do intra-articular corticosteroid injections for osteoarthritis delay the need for joint replacement?	70	77	83	79	57	76	62	75
What are patients’ experiences of having intra-articular corticosteroid injections for osteoarthritis?	74	74	75	88	70	57	76	70
What is the best dose of intra-articular corticosteroid injections to use for patients with osteoarthritis?	71	74	85	88	64	67	57	60
What type of corticosteroid works the best for osteoarthritis?	70	74	89	91	70	57	38	65
Do intra-articular corticosteroid injections help people with osteoarthritis to do exercises/physiotherapy?	75	72	84	85	70	52	67	70
How long do the effects of an intra-articular corticosteroid injections for osteoarthritis last?	69	–	79	–	61	–	62	–
Do the benefits of intra-articular corticosteroid injections for osteoarthritis change with repeated use?	69	–	73	–	78	–	52	–
What is the best time interval between repeated intra-articular corticosteroid injections for osteoarthritis?	64	–	72	–	70	–	43	–
What are the risks of intra-articular corticosteroid injections for osteoarthritis?	63	–	63	–	70	–	57	–
What are patients’ expectations of intra-articular corticosteroid injections for osteoarthritis?	63	–	81	–	52	–	43	–
Does the effect of intra-articular corticosteroid injections for osteoarthritis vary by joint?	59	–	70	–	43	–	57	–
What information should be provided to patients about intra-articular corticosteroid injections for osteoarthritis?	58	–	73	–	43	–	48	–
How long after an intra-articular corticosteroid injection for osteoarthritis can a joint replacement operation be performed?	58	–	57	–	61	–	57	–
What intra-articular corticosteroid injection technique works the best for osteoarthritis?	56	–	86	–	43	–	19	–
What follow-up should be offered to patients after intra-articular corticosteroid injections for osteoarthritis?	54	–	83	–	35	–	29	–
Is there fair access for patients to intra-articular corticosteroid injections for osteoarthritis?	53	–	80	–	35	–	29	–
Should intra-articular corticosteroid injections for osteoarthritis be given in primary care or in a hospital setting?	37	–	51	–	22	–	29	–

R2 = Round 2; R3 = Round 3, - = not retained and carried forward to Round 3.

**Table 4:** Research question priority list for intra-articular corticosteroid injections for osteoarthritis.

What are the long-term effects of repeated intra-articular corticosteroid injections for osteoarthritis? What outcomes are important to patients having intra-articular corticosteroid injections for osteoarthritis? Are intra-articular corticosteroid injections as good as other non-surgical treat- ments at reducing osteoarthritis symptoms? Can the duration of benefit from intra-articular corticosteroid injections for oste-oarthritis be increased? When in the osteoarthritis treatment pathway should patients be offered intra- articular corticosteroid injections? How many intra-articular corticosteroid injections is it safe for patients with oste- oarthritis to receive? Do intra-articular corticosteroid injections reduce osteoarthritis symptoms? Are intra-articular corticosteroid injections for osteoarthritis good value for money for the NHS? Is it possible to predict which patients are most likely to benefit from intra-articular corticosteroid injections for osteoarthritis? Do intra-articular corticosteroid injections for osteoarthritis delay the need for joint replacement? What are patients’ experiences of having intra-articular corticosteroid injections for osteoarthritis? What is the best dose of intra-articular corticosteroid injections to use for patients with osteoarthritis? What type of corticosteroid works the best for osteoarthritis? Do intra-articular corticosteroid injections help people with osteoarthritis to do exercises/physiotherapy?

## Data Availability

The datasets generated during the current study will be available in the University of Bristol Research Data Repository (https://data.bris.ac.uk/data/). Data will be available within 6 months of the publication of the results. Access to the data will be restricted to ensure that data is only made available to bona fide researchers for ethically approved research projects, on the understanding that confidentiality will be maintained and after a Data Access Agreement has been signed by an institutional signatory.
