# Supplementary material for: Research priorities for intra-articular corticosteroid injections for osteoarthritis: A Delphi study
Source: Osteoarthr Cartil Open. 2022 Jun 24;4(3):100291. doi: 10.1016/j.ocarto.2022.100291 (PMC7613692; doi:10.1016/j.ocarto.2022.100291)
Supplement: Multimedia component 1 [file mmc1.docx]

**Supplementary materials**

Contents

[Table 1: Suggestions in Round 1 deemed beyond the scope of the study or not a research question 2](#_Toc98139723)

[Table 2: Candidate research questions developed from Round 1 questionnaire 3](#_Toc98139724)

[Figure 1: Search strategy for literature review 5](#_Toc98139725)

[Table 3: Candidate research questions and volume of existing evidence 6](#_Toc98139726)

# Table 1: Suggestions in Round 1 deemed beyond the scope of the study or not a research question

| **Reasons for exclusion** | **Number** | **Examples** |
| --- | --- | --- |
| Not a research question | 16 | Description of osteoarthritis pain or experiences of receiving injections  Only give injections if necessary  Exercise is beneficial for osteoarthritis  Research into treatments is vital |
| Beyond scope of this study | 9 | Lifestyle factors of patients who require corticosteroid injections  Evaluation of corticosteroid injections for conditions other than osteoarthritis e.g. tennis elbow, trochanteric bursitis, carpal tunnel syndrome  Reasons why NICE guidelines are not taken up in practice  Investigation of alternative reasons for pain |

# Table 2: Candidate research questions developed from Round 1 questionnaire

|  | Candidate research questions | Topic code | No. of times suggested by participants | Patient panel | Health professional panel | Academic panel |
| --- | --- | --- | --- | --- | --- | --- |
| 1 | Do intra-articular corticosteroid steroid injections reduce osteoarthritis symptoms? | Clinical effectiveness | 35 | 14 | 4 | 17 |
| 2 | What are the risks of intra-articular corticosteroid steroid injections for osteoarthritis? | Risks | 33 | 7 | 11 | 15 |
| 3 | Is it possible to predict which patients are most likely to benefit from intra-articular corticosteroid injections for osteoarthritis? | Identifying responders | 24 | 6 | 9 | 9 |
| 4 | Are intra-articular corticosteroid injections as good as other non-surgical treatments at reducing osteoarthritis symptoms? | Alternatives | 21 | 8 | 7 | 6 |
| 5 | How many intra-articular corticosteroid injections is it safe for patients with osteoarthritis to receive? | Repeat injections – number | 18 | 10 | 7 | 1 |
| 6 | When in the osteoarthritis treatment pathway should patients be offered intra-articular corticosteroid injections? | Care pathway | 16 | 5 | 3 | 8 |
| 7 | What information should be provided to patients about intra-articular corticosteroid injections for osteoarthritis? | Information for patients | 13 | 12 | 1 | 0 |
| 8 | How long do the effects of an intra-articular corticosteroid injections for osteoarthritis last? | Duration of benefit | 11 | 6 | 2 | 3 |
| 9 | What intra-articular corticosteroid injection technique works the best for osteoarthritis? | Technique | 10 | 3 | 5 | 2 |
| 10 | Do intra-articular corticosteroid injections help people with osteoarthritis to do exercises/physiotherapy? | Physiotherapy | 10 | 1 | 3 | 6 |
| 11 | Does the effect of intra-articular corticosteroid injections for osteoarthritis vary by joint? | Joint | 9 | 6 | 2 | 1 |
| 12 | What is the best dose of intra-articular corticosteroid injections to use for patients with osteoarthritis? | Dose | 9 | 6 | 3 | 0 |
| 13 | Are intra-articular corticosteroid injections for osteoarthritis good value for money for the NHS? | Cost-effectiveness | 8 | 0 | 2 | 6 |
| 14 | What are the long-term effects of repeated intra-articular corticosteroid injections for osteoarthritis? | Repeat injections – safety | 8 | 2 | 4 | 2 |
| 15 | Should intra-articular corticosteroid injections for osteoarthritis be given in primary care or in a hospital setting? | Primary/secondary care | 8 | 2 | 5 | 1 |
| 16 | What are patients' experiences of having intra-articular corticosteroid injections for osteoarthritis? | Experience | 6 | 2 | 3 | 1 |
| 17 | Do intra-articular corticosteroid injections for osteoarthritis delay the need for joint replacement? | Joint replacement | 6 | 3 | 1 | 2 |
| 18 | What follow-up should be offered to patients after intra-articular corticosteroid injections for osteoarthritis? | Follow-up | 6 | 6 | 0 | 0 |
| 19 | How long after an intra-articular corticosteroid injection for osteoarthritis can a joint replacement operation be performed? | Safety – joint replacement | 5 | 0 | 1 | 4 |
| 20 | Can the duration of benefit from intra-articular corticosteroid injections for osteoarthritis be increased? | Increased duration of benefit | 5 | 5 | 0 | 0 |
| 21 | Do the benefits of intra-articular corticosteroid injections for osteoarthritis change with repeated use? | Repeat injections – change in benefit | 5 | 3 | 1 | 1 |
| 22 | What outcomes are important to patients having intra-articular corticosteroid injections for osteoarthritis? | Outcomes | 5 | 1 | 1 | 3 |
| 23 | What type of corticosteroid works the best for osteoarthritis? | Steroid type | 4 | 0 | 3 | 1 |
| 24 | What is the best time interval between repeated intra-articular corticosteroid injections for osteoarthritis? | Repeat injections – time interval | 4 | 2 | 2 | 0 |
| 25 | What are patients' expectations of intra-articular corticosteroid injections for osteoarthritis? | Expectations | 4 | 0 | 2 | 2 |
| 26 | Is there fair access for patients to intra-articular corticosteroid injections for osteoarthritis? | Access | 2 | 1 | 0 | 1 |

# Figure 1: Search strategy for literature review

# Database Search term Results

1 EMBASE (adult*).ti,ab 1711236

2 EMBASE exp ADULT/ 8783025

3 EMBASE (1 OR 2) 9421827

4 EMBASE (osteoarthriti*).ti,ab 100118

5 EMBASE (osteo-arthriti*).ti,ab 470

6 EMBASE (joint arthriti*).ti,ab 1226

7 EMBASE (bon* arthriti*).ti,ab 31

8 EMBASE (arthriti*).ti,ab 267575

9 EMBASE exp ARTHRITIS/ OR exp "DEGENERATIVE DISEASE"/ OR exp OSTEOARTHROPATHY/ 953532

10 EMBASE (4 OR 5 OR 6 OR 7 OR 8 OR 9) 1002327

11 EMBASE (inject*).ti,ab 994966

12 EMBASE (joint inject*).ti,ab 1640

13 EMBASE (arthrocentes*).ti,ab 1528

14 EMBASE (intraarticul*).ti,ab 8171

15 EMBASE (intra-articul*).ti,ab 20536

16 EMBASE (intra* articul*).ti,ab 20560

17 EMBASE exp "INTRAARTICULAR DRUG ADMINISTRATION"/ 6564

18 EMBASE (11 OR 12 OR 13 OR 14 OR 15 OR 16 OR 17) 1013305

19 EMBASE (steroid*).ti,ab 329548

20 EMBASE (corticosteroid*).ti,ab 156240

21 EMBASE (cortico-steroid*).ti,ab 391

22 EMBASE (cortico*).ti,ab 253080

23 EMBASE (glucocortico*).ti,ab 95934

24 EMBASE (methylpred*).ti,ab 27733

25 EMBASE (methyl-pred*).ti,ab 1918

26 EMBASE (methyl* pred*).ti,ab 2388

27 EMBASE (triamcinolon*).ti,ab 10014

28 EMBASE exp CORTICOSTEROID/ OR exp GLUCOCORTICOID/ 949520

29 EMBASE exp PREDNISOLONE/ 127326

30 EMBASE exp STEROID/ OR exp "FLUORINATED STEROID"/ 1533348

31 EMBASE exp HYDROXYCORTICOSTEROID/ 307

32 EMBASE (19 OR 20 OR 21 OR 22 OR 23 OR 24 OR 25 OR 26 OR 27 OR 28 OR 29 OR 30 OR 31) 1718570

33 EMBASE (18 AND 32) 101247

34 EMBASE (3 AND 10 AND 33) 3993

35 EMBASE (rheumat*).ti,ab 274125

36 EMBASE exp "RHEUMATIC DISEASE"/ 250138

37 EMBASE (35 OR 36) 340633

38 EMBASE 34 NOT 37 2301

# Table 3: Candidate research questions and volume of existing evidence

| **Research question** | **Volume of evidence*** | **Fully answered?** | **Justification** |
| --- | --- | --- | --- |
| Do intra-articular corticosteroid steroid injections reduce osteoarthritis symptoms? |  | No | Mostly small randomised and non-randomised studies in knee osteoarthritis, conflicting conclusions from systematic reviews and meta-analysis of randomised trials |
| What are the risks of intra-articular corticosteroid steroid injections for osteoarthritis? |  | No | Mostly hip or knee osteoarthritis, often limited in scope of safety outcomes evaluated, focus on risk of infection after joint replacement surgery |
| Is it possible to predict which patients are most likely to benefit from intra-articular corticosteroid injections for osteoarthritis? |  | No | Mostly small randomised and non-randomised studies in knee osteoarthritis, commonly comparing corticosteroid injections to other intra-articular injections e.g. platelet-rich plasma or hyaluronic acid |
| Are intra-articular corticosteroid injections as good as other non-surgical treatments at reducing osteoarthritis symptoms? |  | No | Small studies looking at limited number and range of risk factors, mostly in knee osteoarthritis. |
| How many intra-articular corticosteroid injections is it safe for patients with osteoarthritis to receive? |  | No | Lack of research |
| When in the osteoarthritis treatment pathway should patients be offered intra-articular corticosteroid injections? |  | No | Lack of research |
| What information should be provided to patients about intra-articular corticosteroid injections for osteoarthritis? |  | No | Lack of research |
| How long do the effects of an intra-articular corticosteroid injections for osteoarthritis last? |  | No | Lack of research |
| What intra-articular corticosteroid injection technique works the best for osteoarthritis? |  | No | Lack of research |
| Do intra-articular corticosteroid injections help people with osteoarthritis to do exercises/physiotherapy? |  | No | Lack of research |
| Does the effect of intra-articular corticosteroid injections for osteoarthritis vary by joint? |  | No | Lack of research |
| What is the best dose of intra-articular corticosteroid injections to use for patients with osteoarthritis? |  | No | Lack of research |
| Are intra-articular corticosteroid injections for osteoarthritis good value for money for the NHS? |  | No | Lack of research |
| What are the long-term effects of repeated intra-articular corticosteroid injections for osteoarthritis? |  | No | Lack of research |
| Should intra-articular corticosteroid injections for osteoarthritis be given in primary care or in a hospital setting? |  | No | Lack of research |
| What are patients' experiences of having intra-articular corticosteroid injections for osteoarthritis? |  | No | Lack of research |
| Do intra-articular corticosteroid injections for osteoarthritis delay the need for joint replacement? |  | No | Lack of research |
| What follow-up should be offered to patients after intra-articular corticosteroid injections for osteoarthritis? |  | No | Lack of research |
| How long after an intra-articular corticosteroid injection for osteoarthritis can a joint replacement operation be performed? |  | No | Lack of research |
| Can the duration of benefit from intra-articular corticosteroid injections for osteoarthritis be increased? |  | No | Lack of research |
| Do the benefits of intra-articular corticosteroid injections for osteoarthritis change with repeated use? |  | No | Lack of research |
| What outcomes are important to patients having intra-articular corticosteroid injections for osteoarthritis? |  | No | Lack of research |
| What type of corticosteroid works the best for osteoarthritis? |  | No | Lack of research |
| What is the best time interval between repeated intra-articular corticosteroid injections for osteoarthritis? |  | No | Lack of research |
| What are patients' expectations of intra-articular corticosteroid injections for osteoarthritis? |  | No | Lack of research |
| Is there fair access for patients to intra-articular corticosteroid injections for osteoarthritis? |  | No | Lack of research |

*The volume of studies identified that addressed each research question is categorised as ‘none/low (red, <10 studies)’, ‘some’ (orange, 10-30 studies) and ‘high’ (green, >30 studies).
